# Attenuation of Dupuytren’s fibrosis via targeting of the STAT1 modulated IL-13Rα1 response

**DOI:** 10.1126/sciadv.aaz8272

**Published:** 2020-07-10

**Authors:** Moeed Akbar, Emma Garcia-Melchor, Sabarinadh Chilaka, Kevin J. Little, Shatakshi Sood, James H. Reilly, Foo Y. Liew, Iain B. McInnes, Neal L. Millar

**Affiliations:** 1 Institute of Infection, Immunity and Inflammation, College of Medicine, Veterinary and Life Sciences University of Glasgow, Glasgow, Scotland, UK.; 2 Department of Orthopaedic Surgery, Queen Elizabeth University Hospital, Govan Road, Glasgow, Scotland, UK.; 3 School of Biology and Basic Medical Sciences, Soochow University, Suzhou 215006, JS, China.

## Abstract

Fibrotic disorders represent common complex disease pathologies that are therapeutically challenging. Inflammation is associated with numerous fibrotic pathogeneses; however, its role in the multifaceted mechanisms of fibrosis remains unclear. IL-13 is implicated in aberrant responses involved in fibrotic disease, and we aimed to understand its role in the inflammatory processes of a common fibrotic disorder, Dupuytren’s disease. We demonstrated T-cells produced IFN-g, which induced IL-13 secretion from mast cells and up-regulated IL-13Ra1 on fibroblasts, rendering them more reactive to IL-13. Consequently, diseased myofibroblasts demonstrated enhanced fibroproliferative effects upon IL-13 stimulation. We established IFN-g and IL-13 responses involved STAT dependent pathways, and STAT targeting (tofacitinib) could inhibit IL-13 production from mast cells, IL-13Ra1 up-regulation in fibroblasts and fibroproliferative effects of IL-13 on diseased myofibroblasts. Accordingly, utilizing Dupuytren’s as an accessible human model of fibrosis, we propose targeting STAT pathways may offer previously unidentified therapeutic approaches in the management of fibrotic disease.

## Teaser

Targeting STAT pathways may offer previously unidentified approaches in the management of fibrotic disorders.

## INTRODUCTION

Fibrosis is a complex process of aberrant tissue healing, leading to loss of physiological tissue structure and function with inflammatory processes playing a critical role in disease chronicity. Dupuytren's disease of the hand is a classic example of pathological inflammatory fibrosis resulting in a debilitating disorder with a prevalence >7% in the United States, and thus, is commonly encountered by surgeon, physician, and primary care practitioners. Dupuytren’s disease generally begins with nodule formation in the palm of the hand, progressing toward formation of a fibrotic cord toward the fingers, and eventually results in contraction and the loss of ability to extend the digits (*1*, *2*). Presently, the most common treatment for established contractures remains surgical intervention, while the use of collagenase injections has offered an alternative to surgical excisions; however, patients often have notable residual dysfunction due to irreversible fixed flexion deformities of the joints despite these treatments. While meticulous mechanistic investigation of inflammatory pathways utilizing the “molecule to clinical” intervention paradigm has shown remarkable success in other areas of musculoskeletal therapeutics (*3*), there remains no specific disease-modifying treatment for early disease or prevention of recurrence in Dupuytren’s disease.

Inflammation is implicated in numerous fibrotic disorders, and immune cells produce many cytokines, including transforming growth factor–β (TGF-β), that are pivotal (*4*–*7*). The presence of lymphocytes and macrophages along with a number of cytokines [tumor necrosis factor–α (TNF-α), interferon-γ (IFN-γ), interleukin-1β (IL-1β), IL-6, and IL-33] has previously been documented in Dupuytren’s tissue (*2*, *8*–*11*). The main cell responsible for matrix deposition and contraction in Dupuytren's disease has been characterized as alpha smooth muscle actin (α-SMA)–rich myofibroblasts (*2*, *10*, *12*, *13*). The initiating factors associated within normal fascia fibroblasts-to-myofibroblast transdifferentiation in Dupuytren’s disease remain unclear; however, a number of chemical and mechanical elements have been described as playing a role in this phenomenon (*14*). One such chemical factor that has been extensively studied in Dupuytren’s disease is TGF-β, and studies have consistently demonstrated up-regulation of TGF-β in Dupuytren’s disease (*2*, *9*, *12*). Furthermore, TGF-β is a profibrotic cytokine that not only up-regulates α-SMA but also promotes fibroblast proliferation and extracellular matrix (ECM) production deposition (*12*, *15*). In a similar vein, IL-13 is a pleiotropic cytokine produced by a gene on chromosome 5 at q31 that is elaborated in substantial quantities by appropriately stimulated T cells (*16*) and is also reported to induce secretion and activation of latent TGF-β (*6*). IL-13 has been implicated in the pathogenesis of hepatic fibrosis, progressive systemic sclerosis, pulmonary fibrosis, and nodular sclerosing Hodgkin's disease (*5*, *17*).

Given that targeting inflammatory pathways has proven encouraging in other fibrotic disorders (*17*, *18*), we investigated the role for IL-13 signaling as a potential therapeutic target in Dupuytren’s disease. Here, we report immune cell–driven IL-13 production and signaling in Dupuytren’s disease, which enhances the fibroproliferative features of the tissue response primarily through increased IL-13Rα1 signaling. By chromatin immunoprecipitation (ChIP), we also show enriched signal transducer and activator of transcription 1 (STAT1) binding at the IL-13Rα1 site that drive the enhanced fibrotic response in Dupuytren’s disease. We demonstrate manipulation of IL-13 signaling and response pathways via small-molecule inhibition {pan c-Jun N-terminal kinases [Janus kinase (JAK)] inhibitor} of STAT phosphorylation as a possible therapeutic target to down regulate myofibroblast differentiation and activity in Dupuytren’s disease.

## RESULTS

### IL-13 secretion from mast cells in Dupuytren’s disease

**Fig. 1:**
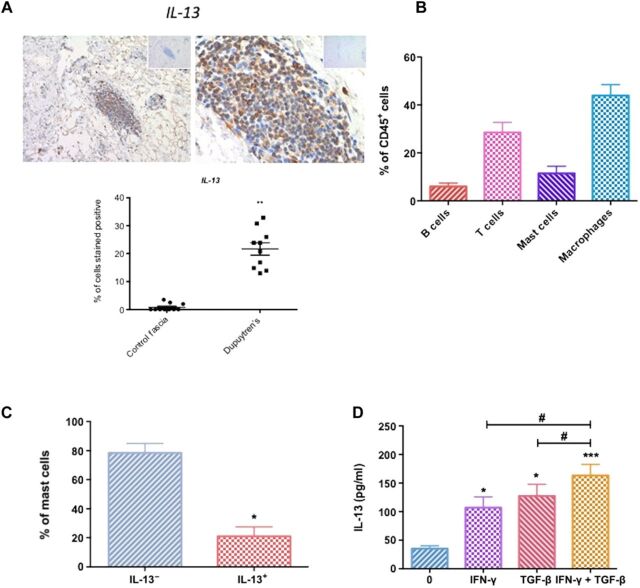
Mast cells produce IL-13 in Dupuytren’s disease. (**A**) Dupuytren’s tissue stained for IL-13, isotype immunoglobulin G (IgG) in the top right corner, using rabbit polyclonal IL-13 antibody at ×10 and ×40 magnification. Graph illustrates percentage of cells stained positive for IL-13, means ± SEM, *n* = 10 for control fascia, *n* = 10 for Dupuytren’s tissue; **P < 0.01. (**B**) Percentage of immune cells phenotyped from disaggregated Dupuytren’s tissue, means ± SEM, *n* = 6. (**C**) IL-13 is produced by mast cells from disaggregated Dupuytren’s tissue. Graph illustrates percentage of IL-13^+^ mast cells, means ± SEM, *n* = 6; *P < 0.01 with null hypothesis 0% of mast cells produce IL-13. (**D**) IL-13 secretion by human buffy coat–derived mast cells following IFN-γ and/or TGF-β treatment; results are means ± SEM, *n* = 6; *P < 0.05, **P < 0.01, ***P < 0.001, significant difference from untreated cells. ^#^*P* < 0.05.

Immunohistochemistry of tissue sections from Dupuytren’s tissue and control fascia demonstrated greater number of cells positively stained for IL-13 (up 30% of cells) in diseased tissue sections (Fig. 1A); this was in an addition to a greater cellularity in Dupuytren’s tissue compared with control fascia, which appeared primarily acellular (fig. S1A). IL-13 can be released from a number of immune cells such as macrophages, mast cells, and T cells (*4*, *19*–*22*). The majority of CD45^+^ immune cells were macrophages with a smaller percentage of mast cells alongside lymphocytes (CD3^+^ T cells and CD19^+^ B cells) (Fig. 1B). We subsequently sought to stimulate cells released from disaggregated Dupuytren’s tissue in vitro to identify IL-13^+^ cells. We found no evidence that macrophages (CD64^+^ macrophages) or T cells were able to secrete IL-13 (fig. S1, B and C). In addition, T cells from diseased tissue were mainly IFN-γ^+^–producing T cells following ex vivo stimulation (fig. S1D). A proportion (mean, 21.01 ± 6.53%) of CD117^+^ mast cells did express IL-13, suggesting that mast cells are the primary source of IL-13 in Dupuytren’s disease (Fig. 1C). We confirmed that exposure to IFN-γ and TGF-β in vitro significantly (*P* < 0.05) increased the production of IL-13 from mast cells as previously shown (*23*, *24*)(Fig. 1D). Together, these data suggest IL-13 is predominantly released from mast cells in Dupuytren’s disease following cytokine exposure.

### IL-13 drives aberrant fibrotic response in Dupuytren’s disease

**Fig. 2:**
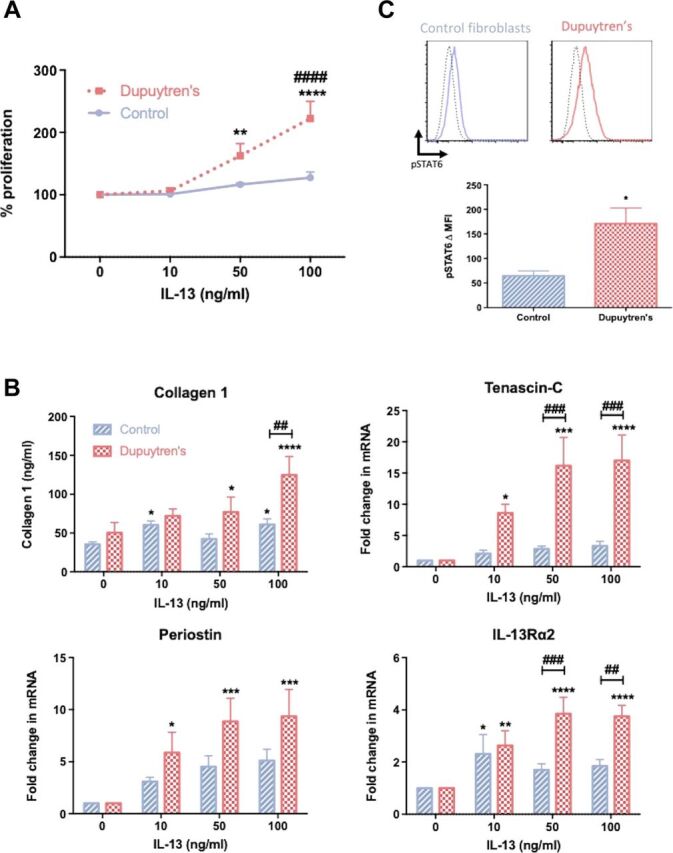
IL-13 drives fibrosis in vitro. (**A**) Effect of recombinant IL-13 on control fibroblast and Dupuytren’s myofibroblast proliferation, means ± SEM, *n* = 6; * indicates significant difference from untreated cells, **P < 0.01, ****P < 0.001; # indicates significant difference from control fibroblasts, ^####^*P* < 0.0001. (**B**) Effect of IL-13 on collagen 1 production and tenascin-C, periostin, and IL-13Rα2 gene expression; mRNA gene expression expressed as fold change following normalization to housekeeping gene [glyceraldehyde-3-phosphate dehydrogenase (GAPDH)] and then to relevant untreated cells, *n* = 6, * indicates significant difference from untreated cells, *P < 0.05, **P < 0.01, ***P < 0.001, ****P < 0.0001; # indicates significant difference from control fibroblasts, ^##^*P* < 0.01, ^###^*P* < 0.001. (**C**) Phosphorylation of STAT6 following IL-13 exposure in control fibroblasts and Dupuytren’s myofibroblasts. Flow cytometric histograms are representative of unstimulated (broken line) and IL-13 stimulated (solid line). Graph demonstrates the change in MFI after IL-13 stimulation, means ± SEM, *n* = 6; *P < 0.05. MFI, median fluorescence intensity.

We next investigated the effect of IL-13 on cell proliferation of diseased and normal fibroblasts. IL-13 significantly (*P* < 0.01) increased proliferation of Dupuytren’s myofibroblasts compared with untreated control cells (Fig. 2A), demonstrating that diseased myofibroblasts proliferate at a greater rate compared with control fibroblasts following IL-13 exposure. Dupuytren’s diseased is characterized by highly proliferating α-SMA–expressing myofibroblasts. Concordantly, Dupuytren’s myofibroblasts had significantly (*P* < 0.05) greater α-SMA mRNA expression compared with control fibroblasts (fig. S2A). However, no response in α-SMA mRNA expression was observed in response to IL-13 stimulation in vitro. As one of the main hallmarks of Dupuytren’s disease is dysregulated matrix deposition, particularly collagen (*12*, *25*), and as IL-13 is known to directly affect matrix protein production (*26*), we sought to determine the effect of IL-13 on matrix gene expression in both control and diseased cells. We observed that collagen 1 production was increased in both control and diseased cell following IL-13 exposure (Fig. 2B). However, we also noted a greater level of increase in collagen 1 production by Dupuytren’s myofibroblasts compared with control fibroblasts following IL-13 treatment, particularly at higher doses of IL-13. Furthermore, the mRNA expression of matrix proteins tenascin-C and periostin significantly (*P* < 0.05) increased to a greater degree in Dupuytren’s myofibroblasts compared with control fibroblasts following exposure to IL-13 (Fig. 2B) in vitro. To further assess whether IL-13 had a greater effect on diseased myofibroblasts, we measured the expression of IL-13Rα2, the decoy receptor, which is directly responsive to IL-13 (*27*). We observed that the increase in IL-13Rα2 gene expression was significantly (*P* < 0.01) greater in diseased cells following exposure to IL-13 in vitro.

As IL-13 signaling is primarily via the phosphorylation of STAT6 (*28*), utilizing an antibody specific against pSTAT6 (phosphorylated STAT6), we confirmed significantly (*P* < 0.05) greater pSTAT6 in diseased cells versus control fibroblasts after IL-13 stimulation (Fig. 2C). This confirmed enhanced IL-13 signaling in Dupuytren’s myofibroblasts compared with control fibroblasts.

### Enhanced IL13Rα1 signaling in Dupuytren’s fibroblasts

**Fig. 3:**
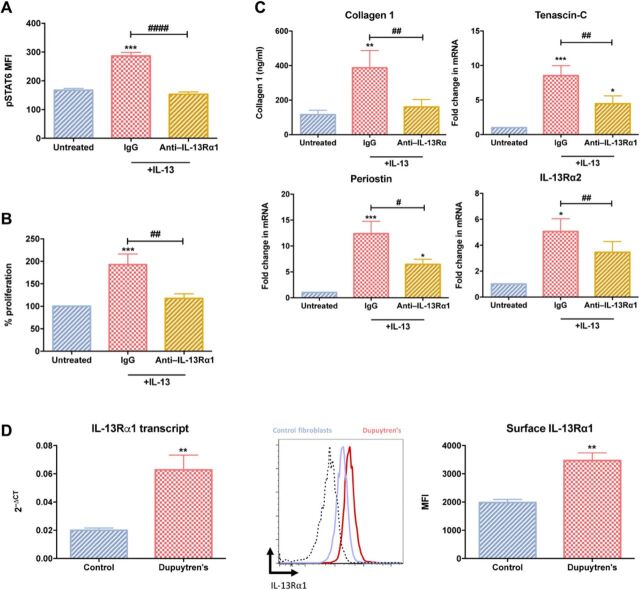
Enhanced IL-13 signaling through IL-13Rα1 in Dupuytren’s myofibroblasts in vitro. Dupuytren’s myofibroblasts were pretreated with anti–IL13Rα1 before exposure to IL-13 (100 ng/ml). (**A**) Phosphorylation of STAT6. (**B**) Proliferation of Dupuytren’s myofibroblasts. (**C**) Effect on collagen 1 production and tenascin-C, periostin, and IL-13Rα2 gene expression. mRNA gene expression expressed as fold change following normalization to housekeeping gene (GAPDH) and then to relevant untreated cells. All results are means ± SEM, *n* = 6; * indicates significant difference from untreated cells, *P < 0.05, **P < 0.01, ***P < 0.001. ^#^*P* < 0.05, ^##^*P* < 0.01, ^###^*P* < 0.001. (**D**) IL-13Rα1 protein and transcript expression in control fibroblasts and Dupuytren’s myofibroblasts. Representative flow cytometry histogram of control fibroblasts (blue line) and Dupuytren’s myofibroblasts (red line). Graphs illustrates MFI or 2^−ΔCT^ (relative to GAPDH) of IL-13Rα1, means ± SEM, *n* = 6; **P < 0.01.

As both IL-13 signaling and its downstream effects were increased in diseased myofibroblasts, we next explored the receptor responsible for enhanced IL-13 signaling. We confirmed that IL-13 signaling in diseased myofibroblasts was primarily through IL-13Rα1 by pretreating myofibroblasts with an IL-13Rα1–blocking antibody before IL-13 exposure. This resulted in a complete amelioration of IL-13–induced STAT6 phosphorylation, ECM protein production, and cell proliferation (Fig. 3, A to C). This demonstrated IL-13 signaled through IL-13Rα1, inducing phosphorylation of STAT6. By negating IL-13 signaling through blocking of the signaling receptor, we were able to prevent myofibroblast proliferation and, in particular, inhibit the IL-13–stimulated ECM production: collagen I, tenascin-C, and periostin. We observed increased (*P* < 0.01) IL-13Rα1 transcript and protein expression in diseased myofibroblasts versus control fibroblasts (Fig. 3D). We found no difference in IL-4 receptor [which IL-13 can also signal through and has been implicated in myofibroblast transdifferentiation (*29*)] expression between diseased and control cells (fig. S2B), suggesting the transdifferentiation of normal fibroblast to myofibroblast results in increased IL-13Rα1 expression and subsequent IL-13 signaling.

### Enriched STAT1 binding at IL-13Rα1 sites drives enhanced fibrotic response in Dupuytren’s disease

**Fig. 4:**
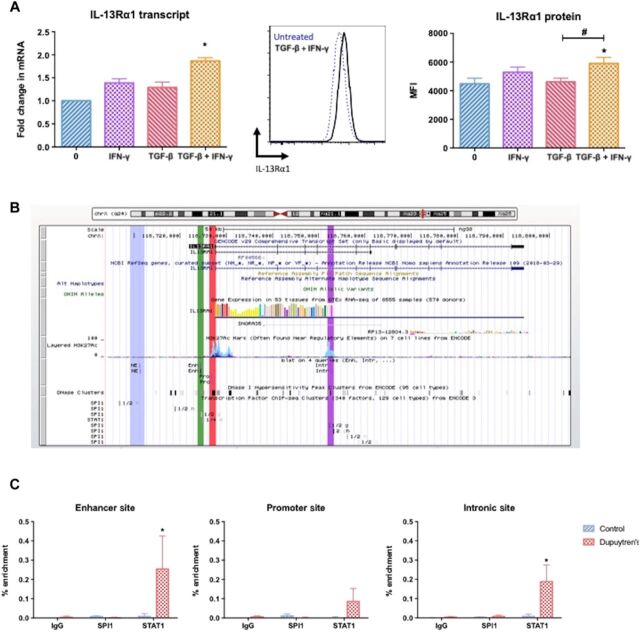
STAT1 mediated dysregulation of IL13Rα1 in Dupuytren’s disease. (**A**) Control fibroblasts exposed to IFN-γ and/or TGF-β. IL-13Rα1 transcript following treatment. Representative flow cytometry histogram of IL-13Rα1 expression on the surface of untreated, and graph illustrates MFI of IL-13Rα1 after treatment. All results are means ± SEM; mRNA gene expression expressed as fold change following normalization to housekeeping gene (GAPDH) and then to relevant untreated cells, *n* = 6; * indicates significant difference from untreated cells, *P < 0.05. ^#^*P* < 0.05. (**B**) Identification of sites of interest on IL-13Rα1 gene locus: nonspecific (blue), enhancer (green), promoter (red), and intronic site (purple). (**C**) Quantitative polymerase chain reaction of ChIP representing percent enriched binding of SPI1 and STAT1 at different sites of interest of interest on the IL-13Rα1 gene. Graph demonstrates the percent enriched following subtraction of nonspecific enrichment, means ± SEM, *n* = 4; *P < 0.05.

As our data demonstrated TGF-β and IFN-γ induced IL-13 release from mast cells, we next examined whether these cytokines could also alter IL-13Rα1 expression in control fibroblasts as had been previously demonstrated in other cells (*30*). While TGF-β exposure alone did not significantly (*P* > 0.05) increase IL-13Rα1 expression, IFN-γ stimulation induced control fibroblasts to up-regulate IL-13Rα1 protein on the cell surface (Fig. 4A). Further to this, treatment of normal fibroblasts with both IFN-γ and TGF-β significantly (*P* < 0.05) increased the surface expression of IL-13Rα1 and its transcript (Fig. 4A).

Having found that IFN-γ (Fig. 4A) and STAT1 signaling (fig. S3A) has a key role in manifesting the enhanced effects of IL-13 via up-regulation of IL-13Rα1 in Dupuytren’s disease, we investigated whether differential STAT1 binding at the IL-13Rα1 gene locus may cause this to enhance its activity by performing ChIP enrichment analysis. Using the publicly available UCSC (University of California Santa Cruz) genome browser database, we identified a number of potential binding sites of interest for STAT1 and SPI1 [a key regulator of myofibroblast differentiation in fibrosis (*31*)] on the IL-13Rα1 gene locus (Fig. 4B). These regions include (i) the promoter (marked red); (ii) a possible STAT1 binding site upstream from the promoter, which may act as an enhancer site (green); and (iii) an intronic region that based on the enriched histone H3K27ac mark is associated to an active gene regulatory element (purple). In addition, an upstream region from the same locus that does not overlap with any of the binding sites was analyzed as a negative control (blue). ChIP analysis with antibodies against STAT1 and SPI1 demonstrated differential STAT1 recruitment at the IL-13Rα1 gene locus between Dupuytren’s myofibroblasts and healthy fibroblasts (Fig. 4C). The results showed an enrichment of STAT1 binding at the upstream enhancer and intronic binding sites in Dupuytren’s myofibroblasts (Fig. 4C). Furthermore, SPI1 ChIP analysis did not show binding at the IL-13Rα1 gene, at the sites examined in either control fibroblasts or diseased myofibroblasts. In summary, these data indicate enhanced STAT1 binding at the IL-13Rα1 gene locus, specifically in Dupuytren’s myofibroblasts, which may be the cause of enhanced receptors’ activity in disease.

### STAT inhibition in Dupuytren’s disease: Translational targeting

**Fig. 5:**
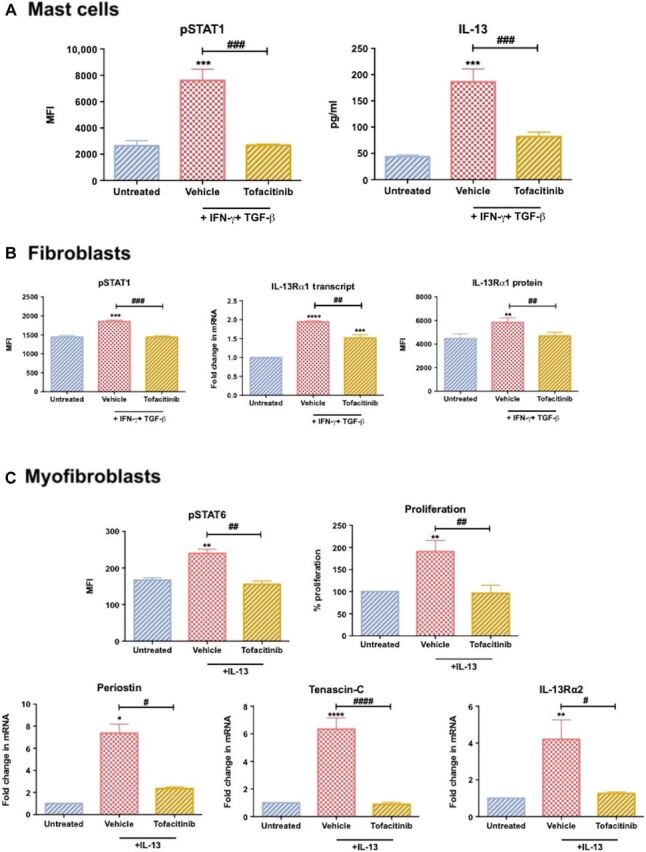
Inhibition of STAT phosphorylation reduces IL-13 secretion and signaling. (**A**) STAT1 phosphorylation and IL-13 secretion from human mast cells following IFN-γ and TGF-β exposure. Cells were pretreated with tofacitinib or vehicle control [0.001% dimethyl sulfoxide (DMSO)] for 30 min. (**B**) STAT1 phosphorylation and IL-13Rα1 transcript and protein expression following IFN-γ and TGF-β treatment. Control fibroblasts were pretreated with tofacitinib or vehicle control (0.001% DMSO) for 30 min before cytokine stimulation. (**C**) Dupuytren’s myofibroblasts were pretreated with tofacitinib or vehicle control (0.001% DMSO) and then exposed to IL-13 (100 ng/ml). STAT6 phosphorylation, myofibroblast proliferation, and periostin, tenascin-C, and IL-13Rα2 transcript levels. All results are means ± SEM; mRNA gene expression expressed as fold change following normalization to housekeeping gene (GAPDH) and then to relevant untreated cells, *n* > 4; * indicates significant difference from untreated cells, *P < 0.05, **P < 0.01, ***P < 0.001, ****P < 0.001. ^#^*P* < 0.05, ^##^*P* < 0.01, ^###^*P* < 0.001, ^####^*P* < 0.0001.

The effect of IFN-γ and IL-13 stimulation is dependent on STAT phosphorylation (*28*, *32*, *33*). We identified IFN-γ–induced IL-13 production in mast cells and IL-13Rα1 expression in fibroblasts are accompanied by increased STAT1 phosphorylation (fig. S2), while IL-13–driven effects in myofibroblasts are accompanied by STAT6 phosphorylation (Fig. 2B). Tofacitinib is a JAK inhibitor, which inhibits both JAK1 and JAK3 that are vital for STAT1 and STAT6 phosphorylation (*34*, *35*). As these pathways are vital for increased IL-13 production and signaling, we explored the use of this inhibitor as a potential therapeutic intervention in Dupuytren’s disease. First, we assessed whether tofacitinib could negate the IFN-γ–induced IL-13 production by mast cells. We observed that STAT1 phosphorylation was significantly (*P* < 0.001) inhibited in cytokine-stimulated mast cells that had been pretreated with tofacitinib compared with control vehicle alone (Fig. 5A). In addition, the data demonstrated that IL-13 produced by cytokine-stimulated mast cells was significantly (*P* < 0.001) lower following tofacitinib pretreatment (Fig. 5A).

We further assessed whether tofacitinib treatment could reduce the observed increase in IL-13Rα1 expression. Initially, we established that control fibroblasts treated with tofacitinib before IFN-γ (+TGF-β) stimulation did not increase pSTAT1 levels, whereas cells pretreated with vehicle alone did (Fig. 5B). Furthermore, IL-13Rα1 transcript and protein levels following IFN-γ plus TGF-β exposure were significantly (*P* < 0.01) lower in cells pretreated with tofacitinib compared with those pretreated with vehicle control. Together, these results demonstrate that STAT1 targeting via tofacitinib is capable of inhibiting IFN-γ–driven IL-13Rα1 up-regulation observed in diseased myofibroblasts. Last, we ascertained whether tofacitinib could inhibit IL-13 signaling in diseased cells. We observed that tofacitinib did indeed inhibit IL-13–driven STAT6 phosphorylation (Fig. 5C) while additionally abolishing the IL-13–driven changes in periostin, tenascin-C, and IL-13Rα2. Last, we established myofibroblasts pretreated with tofacitinib had significantly (*P* < 0.01) lower levels of proliferation following IL-13 exposure than vehicle-pretreated cells. Together, these data suggest STAT inhibition may be a viable intervention of the different fibroproliferative features observed in Dupuytren’s disease.

## DISCUSSION

**Fig. 6:**
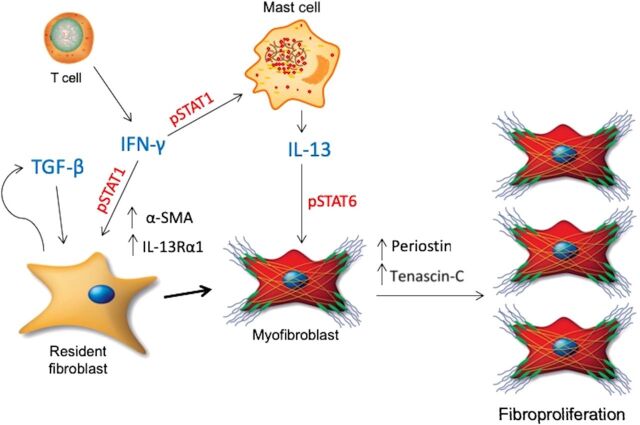
Targeting STAT phosphorylation in Dupuytren’s disease. Schematic diagram illustrating the cytokine-driven milieu in which Dupuytren’s disease may manifest. The presence of IFN-γ–secreting T cells results in IL-13 production from mast cells in addition to fibroblast undergoing myofibroblast transdifferentiation in the presence of TGF-β. These inflammatory interactions drive fibroproliferative remodeling, which can be inhibited by use of STAT inhibitors such as tofacitinib.

This study demonstrates that the local tissue environment in Dupuytren’s disease is characterized by a milieu of inflammatory cells and cytokines, which not only drive early myofibroblast transdifferentiation but also provides the environment for the fibroproliferative chronicity of this debilitating disease. We find a milieu of cytokines drive phenotypic changes on the stromal cells, and these phenomena are dependent on STAT phosphorylation (Fig. 6), which we are able to inhibit in vitro. Together, our results suggest that Dupuytren’s disease has a vital immune and inflammatory component driving the fibroproliferative chronicity that may be effectively treated by targeting the STAT pathway.

IL-13 is synonymous with fibrotic disorders (*19*, *36*, *37*), and we established the increased presence of mast cell–derived IL-13 in Dupuytren’s tissue. One of the principal mechanisms through which IL-13 promotes fibrosis is via proliferation of resident stromal cells. This hyperproliferation, previously attributed in Dupuytren’s disease to TGF-β responses, encourages cells to lay down increased matrix proteins promoting clinical cord and nodule formation. Our data suggest the presence of another core mediator of fibrosis, IL-13, can also induce significant proliferation of myofibroblasts as well as ECM production in Dupuytren’s fibrosis. T cells are also a known source of IL-13 and are found in abundance in Dupuytren’s tissue. However, we identified the majority of T cells in Dupuytren’s tissue were IFN-γ–producing cells. While IFN-γ is not synonymous with fibrosis (*38*, *39*) unlike other cytokines (i.e., IL-4, IL-13, TGF-β), it has also been implicated in fibrotic pathways and pathogenesis (*23*, *24*, *40*–*42*). Similar to previous publications (*23*, *24*), we found that IFN-γ (in combination with TGF-β) was able to induce human mast cells to produce IL-13. We believe that IFN-γ from T cells can induce mast cells to release IL-13 with subsequent fibroproliferative changes observed in disease. The data highlight a milieu of cytokines act cooperatively to induce the multicellular fibrotic pathogenesis observed in Dupuytren’s disease. Rather than being a linear hierarchical pathway, it is likely a dynamic fluid environment leads to the multifaceted features of fibrotic disorders (Fig. 6). Recent findings also support the role of cytokine and cell cross-talk in driving fibrotic disease (*11*). Both macrophages and, in particular, mast cell are synonymous with fibrotic disease, which is further elegantly highlighted by the study. Although the study primarily focused on the combined effects of TNF-α and IL-33, we believe it supports our findings. Izadi and colleagues did not record much spontaneous IL-13 release (*11*), which may be due to cells being latent following disaggregation from tissue; IL-33 is known to drive IL-13 release from mast cells in disease (*43*). The current study has demonstrated that IL-13 release and its downstream fibrotic effects are viable targets for therapy. As such, the findings from both studies reinforce that fibrotic disease is a result of a complex milieu of cells and cytokines working cooperatively in the pathogenesis of fibrosis.

Our data further support the notion that α-SMA–high–expressing myofibroblast phenotype is primarily due to TGF-β (fig. S3C) but reveal Dupuytren’s myofibroblasts portray another, to our knowledge, undocumented facet: higher IL-13Rα1 expression. In addition, we were able to induce this characteristic in control fibroblasts following IFN-γ exposure in combination with TGF-β, a phenomenon previously demonstrated in eosinophils (*30*). As in most cases with IFN-γ (*32*), the induced IL-13Rα1 change was dependent on the phosphorylation of STAT1. We importantly found increased STAT1 binding on the IL-13Rα1 gene at the enhancer and intronic sites, thereby facilitating the downstream IL-13 responsiveness. This unique binding motif of STAT1 appears to be a hallmark of Dupuytren’s myofibroblasts and could represent an interesting therapeutic intervention in the future via epigenetic targeting.

The STAT pathway has been a therapeutic approach in other disease pathologies through the use of JAK inhibitors (*44*, *45*), a group of receptor-associated kinases that are essential for downstream signaling cascade of a number of cytokine receptors. Upon cytokine and receptor engagement, JAKs initiate the STAT phosphorylation, leading to STAT dimerization and, ultimately, target gene induction (*44*). We elected to use tofacitinib as a pan JAK inhibitor, as it has previously been shown to be effective against IFN-γ (*46*) and IL-13 (*35*) signaling in vitro, and safety and efficacy have been demonstrated in numerous clinical trials (*44*) including the treatment of rheumatoid arthritis. We successfully inhibited STAT1 phosphorylation, following IFN-γ stimulation, in mast cells and control fibroblasts, resulting in no downstream increase in IL-13 secretion and IL13Rα1 expression, respectively. Furthermore, tofacitinib treatment inhibited IL-13-induced STAT6 phosphorylation in diseased myofibroblasts, leading to reduced cell proliferation and matricellular protein up-regulation. Together, we demonstrate that targeting of the JAK/STAT pathway can inhibit cytokine production, fibroblast-to-myofibroblast transdifferentiation, and fibroproliferation of myofibroblasts, advocating it as a translational target in Dupuytren’s disease.

While we demonstrate effective targeting of IL-13 production and signaling, we acknowledge a critical role of TGF-β in driving Dupuytren’s disease. Many of the effects we observed occur in the presence of TGF-β, while myofibroblast transdifferentiation via α-SMA expression is principally TGF-β dependent (*47*). However, direct and universal inhibition of TGF-β may not be suitable given its function in a broad range of physiological pathways and various isoforms (*7*, *48*), while in vivo studies have documented increased inflammation, tumor production, and toxicity (*49*–*51*). In addition, clinical trials have demonstrated limited long-term efficacy in fibrotic disorders to date (*47*). Furthermore, α-SMA expression may only be an indicator of fibrotic conditions and not causative (*52*); thus, direct targeting of it may be futile.

We acknowledge that our experimental datasets lack in vivo animal data. Although a number of animal models have been used to study cytokine-driven fibrotic pathogenesis, there is no well-described in vivo model available for Dupuytren’s disease. There have been attempts to describe rodent models; however, these xenograft models rely on implanting human cells into mice or rats (*53*, *54*). In addition, the outcomes of using these model studies do not document any clinical-like outcomes but rather those already investigated in vitro (i.e., TGF-β levels and α-SMA and collagen expression) (*55*–*57*). In addition, while we were able to measure collagen protein levels following IL-13 stimulation, we measure mRNA expression for other matrix proteins (tenascin-C and periostin). However, this was primarily to highlight that changes in these genes were due directly to IL-13, and any subsequent intervention in the IL-13 pathway would impede changes in these genes. Last, while not statistically significant, we also note that our control fascia samples were on average 7 years younger than that of the Dupuytren’s samples, and thus, some aspects may be due to aging effects.

In summary, the current study establishes inflammation-driven epigenetic changes in fibroblasts and IL-13 production in the pathogenesis of Dupuytren’s disease. Dissection of the molecular pathways involved reveals pharmacological manipulation of the STAT pathways as therapeutic targets to down-regulate myofibroblast differentiation and activity. On the basis of our findings, we suggest that repurposing of these readily available pharmaceutical agents may provide novel proof-of-concept studies in Dupuytren’s disease.

## MATERIALS AND METHODS

### Study design

The aim of this study was to dissect the role of inflammation, particularly cytokines, in the manifestation of a common fibrotic pathology: Dupuytren’s disease. Furthermore, we aimed to identify whether any of the pathways involved could be suitable candidates for therapeutic intervention. We identified the presence of immune cells in Dupuytren’s disease and their ability to secrete a number of cytokines synonymous with fibrosis and inflammation, IL-13 and IFN-γ. Next, we investigated the fibroproliferative effects of IL-13 in vitro and whether there were any differences in control and diseased cells. We then sought to investigate the mechanism by which diseased cells were more responsive to IL-13 stimulation. Last, we wished to identify whether the pathways identified could be inhibited using a currently available pharmacological agent that could inhibit the multiple pathways involved in disease.

### Study approval

Human study procedures and protocols were approved by the National Health Service West of Scotland Ethics Committee (REC 11/S0704/7). Full informed consent was obtained from all patients. Sample size for tissue- and cell-based assays was determined on the basis of sample availability and technical needs.

### Clinical samples

Dupuytren’s samples were collected from patients undergoing surgical fasciectomy for Dupuytren’s contracture (*n* = 10, 5 females, 5 males; mean age ± SEM, 50.3 ± 10.2). Control tissue was obtained from patients undergoing hand surgery for carpal tunnel syndrome unaffected by Dupuytren's disease, characterized as normal palmar fascia tissue (*n* = 10, 5 females, 5 males; mean age ± SEM, 43.3 ± 8.6) macroscopically at the time of surgery and microscopically by hematoxylin and eosin (H&E) staining before any control experiments.

### Histology

Tissue was fixed in 4% paraformaldehyde and embedded in paraffin using standard techniques. Sections (5 μm) were obtained and stained with H&E (Vector Laboratories).

### Immunohistochemistry

Histological sections were deparaffinized in xylene and rehydrated through graded ethanol. Endogenous peroxidase activity was quenched using 3% H_2_O_2_. Antigen retrieval was performed using Uni-Trieve. Blocking of nonspecific binding was performed using 2.5% horse blocking serum (both Vector Laboratories). Sections were incubated overnight at 4°C with the primary antibody against IL-13, clone A130D (LSBio), or isotype control. Staining of antigens was performed using the ImmPRESS and ImmPACT DAB chromagen solution as per the manufacturer’s instructions (Vector Laboratories). Sections were counterstained using hematoxylin.

Tissue analysis occurred in two stages by two independent assessors (N.L.M. and J.H.R.) as previously described (*58*); the first stage had all samples being given a semiquantitative grade based on the percentage of positively stained cells (taken over the total number of cells in that field) in 10 random high-powered fields. The following semiquantitative grading was used: grade 0, no staining; grade 1, mild, ≤10% of cells stained positive; grade 2, moderate, 10 to 20% of cells stained positive; and grade 3, strong, ≥20% of cells stained positive. The mean of these values was analyzed by an unpaired Student’s *t* test. In the second stage, the samples had 10 random high-powered fields analyzed at ×40 magnification, and cells in each field were counted manually. The mean percentage of positively stained cells was taken over the total number of cells per high-powered field; similarly, the results were analyzed by an unpaired Student’s *t* test.

### Ex vivo stimulation

Tissue was digested in Liberase TM (Sigma-Aldrich) in Roswell Park Memorial Institute (RPMI) media for 2 hours at 37°C. The digested tissue was passed through a 100-μm cell strainer, pelleted by centrifugation at 350*g* for 5 min; the supernatant was discarded. This was repeated twice. Cells were either harvested for flow cytometry or underwent the appropriate stimulation for 24 hours listed below before harvesting for intracellular flow cytometry. For mast cell stimulation, the cell suspension was incubated with human myeloma immunoglobulin E (IgE) (2 μg/ml) (Merck) for 24 hours and followed by incubation with human anti-IgE (5 μg/ml) (BioLegend). T cell and macrophage stimulation was assessed by stimulating the disaggregated cell suspension for 24 hours with phorbol 12-myristate 13-acetate (50 ng/ml) plus ionomycin (1 μg/ml) and lipopolysaccharide (100 ng/ml) (all Sigma-Aldrich), respectively.

### Mast cell culture

Human buffy coat–derived mast cells were culture as per previously described (*53*).

### Mast cell stimulation

Buffy coat–derived mast cells were incubated with human myeloma IgE (2 μg/ml) for 24 hours. These cells were then stimulated with TGF-β (2 ng/ml) and IFN-γ (10 ng/ml) in the presence of human anti-IgE (5 μg/ml) (all BioLegend) for 15 min for phosphorylation studies or 24 hours for all other studies.

### Fibroblast and myofibroblast isolation and culture

Control fibroblasts and Dupuytren’s myofibroblasts were extracted from control carpal fascia and Dupuytren’s nodules, respectively. Tissue was disaggregated as described above, and cell suspension was resuspended in supplemented culture medium [RPMI with 10% fetal bovine serum, 1% penicillin/streptomycin, and 1% l-glutamine (all Invitrogen)] at 37°C, 5% CO_2_, and 95% humidity. Cultures were maintained at 37°C in a humidified atmosphere of 5% CO_2_. Cells were subcultured and trypsinized at subconfluency and used at passages 2 to 4.

### In vitro fibroblast and myofibroblast stimulation

Cells were plated in 12-well culture plates at a density of 5 × 10^4^ cells per well with 1 ml of supplemented culture medium and allowed to adhere 48 hours before stimulation. Cells were stimulated with the appropriate recombinant human cytokine diluted in supplemented culture medium for 24 hours unless stated below. The concentrations of cytokines used were as follows: IL-13, 1 to 100 ng/ml; TGF-β, 2 ng/ml; and IFN-γ, 10 ng/ml (all BioLegend). After the stimulation period, supernatants were collected, and cells were harvested for the appropriate assay.

For the cell proliferation assay, cells were serum starved overnight before stimulation. The stimulation period was 72 hours. Cells for the phosphorylation studies were also serum starved overnight before stimulation for 15 min.

### Flow cytometry

Single-cell suspensions were labeled with fluorophore-conjugated anti-mouse antibodies (Table 1) and Zombie viability dye at recommended dilutions following the manufacturers’ recommendations (BioLegend). Where adherent cells were used, cells were first rinsed with phosphate-buffered saline (PBS) and then detached using Accutase (BioLegend) as per the manufacturer’s instructions. For phosphorylation studies, BD Phosflow (BD Biosciences) antibodies and protocols were used as described by the manufacturer. Data were acquired on BD LSR II using FACS (fluorescence-activated cell sorting) DIVA software with automated compensation (BD Biosciences). Compensation data were acquired using single stained BD Comp beads as per the manufacturer’s instructions (BD Biosciences). All data were analyzed using FlowJo software (Tree Star).

**Table 1:** Flow cytometry antibodies used in current study.

**Antibody**	**Cells identified**
CD45	Immune cells
CD3	T cells
CD19	B cells
CD64	Macrophages
CD117	Mast cells
pSTAT6	–
Zombie viability dye	All viable cells

### Gene expression

Cells were harvested in PureLink lysis buffer containing 1% 2-mercaptoethanol, and RNA was extracted using mini columns according to the PureLink protocol (Thermo Fisher Scientific). RNA concentration and purity were determined using a spectrophotometer (Nanodrop 2000, Thermo Fisher Scientific). RNA (100 ng) was converted to cDNA using High-Capacity cDNA Reverse Transcription Kit (Thermo Fisher Scientific) according to the manufacturer’s instructions. cDNA was diluted 1 in 5 using ribonuclease-free water. Quantitative polymerase chain reaction (qPCR) was performed using PowerUp SYBR Green Master Mix (Thermo Fisher Scientific). Each sample was run in duplicate and normalized to endogenous control [glyceraldehyde-3-phosphate dehydrogenase (GAPDH)] following confirmation that there was no more than 0.25 cycle difference in the control gene between each treated condition. Data represent relative mRNA expression (2^−ΔCT^) or fold change from untreated cells (2^−ΔΔCT^).

Primers (Integrated DNA Technologies) were as follows: *GAPDH*, (f) 5′-TCGACAGTCAGCCGCATCTTCTTT-3′ and (r) 5′-ACCAAATCCGTTGACTCCGA CCTT-3′;

*Tenascin-C*, (f) 5′-CTTTGGCTGGGTTGCTTGAC-3′ and (r) 5′-GTGCCAGGAGACCGTACCAC-3′; *Periostin*, (f) 5′-TTG AGA CGC TGG AAG GAA AT-3′ and (r) 5′-AGA TCC GTG AAG GTG GTT TG-3′; IL-13Rα1, (f) 5′-CTTCCCGTGTGAAACCTGAT-3′ and (r) 5′-GTGTCTCAGTTTGGCTGTTATTG-3′; *IL-13R*α*2*, (f) 5′-AGCATACCTTTGGGACCTAT-TC-3′ and (r) 5′-TCAACTGTAGCAGTCACCAAG-3′; α-*SMA*, (f) 5′-CCT CCC TTG AGA AGA GTT ACG A-3′ and (r) 5′-GAC TCC ATC CCG ATG AAG GAT-3′.

### Proliferation assay

Cell proliferation was assessed using the Cell Proliferation Kit I (MTT) as per the manufacturer’s protocol (Sigma-Aldrich).

### Chromatin immunoprecipitation

Cultured cells were washed with PBS and cross-linked with 1% formaldehyde, followed by cell lysis using SDS buffer. Lysate was sonicated with Bioruptor UCD-200 (Diagenode), followed by incubation with Dynabeads (Invitrogen) conjugated with rabbit-derived antibodies: SPI1, STAT1, or isotype control (all Cell Signaling Technology). Protein/DNA/bead complexes were washed with radioimmunoprecipitation assay buffer (RIPA buffer), RIPA + NaCl, LiCl, and TE buffer. Protein/DNA complexes were eluted with elution buffer. Reverse cross-linking was performed overnight at 65°C, followed by DNA purification and qPCR. ChIP signal was normalized to total chromatin input (percent input), which was calculated as 100 × 2^(*C*Tinput-*C*Ttarget)^.

Primers (Integrated DNA Technologies) were as follows: *Promoter*, (f) 5′-FW CAG GAA ACG CCT AAG GAC TC-3′ and (r) 5′-AAGGGATGGGAGGTGAATCG-3′; *Enhancer*, (f) 5′-ATCTCTCTCACCTTTGGCGC-3′ and (r) 5′-ATGCAGTGAGTAGAGGCTGG-3′; *Intronic*, (f) 5′-TCTTGTCTCACGGCACCTTG-3′ and (r) 5′-AAACAAGACACAGCTCCCGC-3′; *Negative Control*, (f) 5′-GGCTACCCAGAGTAATGACC-3′ and (r) 5′-GAAGAAAGGGACATGGCAGC-3′.

### Collagen I assay

Collagen I from culture supernatant was determined using either single-antibody enzyme-linked immunosorbent assay kits (R&D Systems) as per the manufacturer’s instructions.

### Statistical analysis

All data are shown as means ± SEM. All statistical analyses, including Shapiro-Wilk normality test, analysis of variance (ANOVA), Fisher’s least significant difference with Bonferonni correction for multiple comparisons, and Student’s *t* test, were performed using GraphPad Prism 7 software. A *P* value of <0.05 was considered significant.

## Supplementary Material

aaz8272_SM.pdf

## SUPPLEMENTARY MATERIALS

Supplementary material for this article is available at http://advances.sciencemag.org/cgi/content/full/6/28/eaaz8272/DC1

## Appendix

View/request a protocol for this paper from *Bio-protocol*.
