# Supplementary material for: Attenuation of Dupuytren’s fibrosis via targeting of the STAT1 modulated IL-13Rα1 response
Source: Sci Adv. 2020 Jul 10;6(28):eaaz8272. doi: 10.1126/sciadv.aaz8272 (PMC7351483; doi:10.1126/sciadv.aaz8272)
Supplement: aaz8272_SM.pdf [file aaz8272_SM.pdf]

Science Advances
AAAS

advances.sciencemag.org/cgi/content/full/6/28/eaaz8272/DC1

Supplementary Materials for

Attenuation of Dupuytren's fibrosis via targeting of the STAT1 modulated IL-13Rα1 response

Moeed Akbar, Emma Garcia-Melchor, Sabarinadh Chilaka, Kevin J. Little, Shatakshi Sood, James H. Reilly, Foo Y. Liew, Iain B. McInnes, Neal L. Millar*

*Corresponding author. Email: neal.millar@glasgow.ac.uk

Published 10 July 2020, *Sci. Adv.* **6**, eaaz8272 (2020)
DOI: 10.1126/sciadv.aaz8272

This PDF file includes:

Figs. S1 to S3

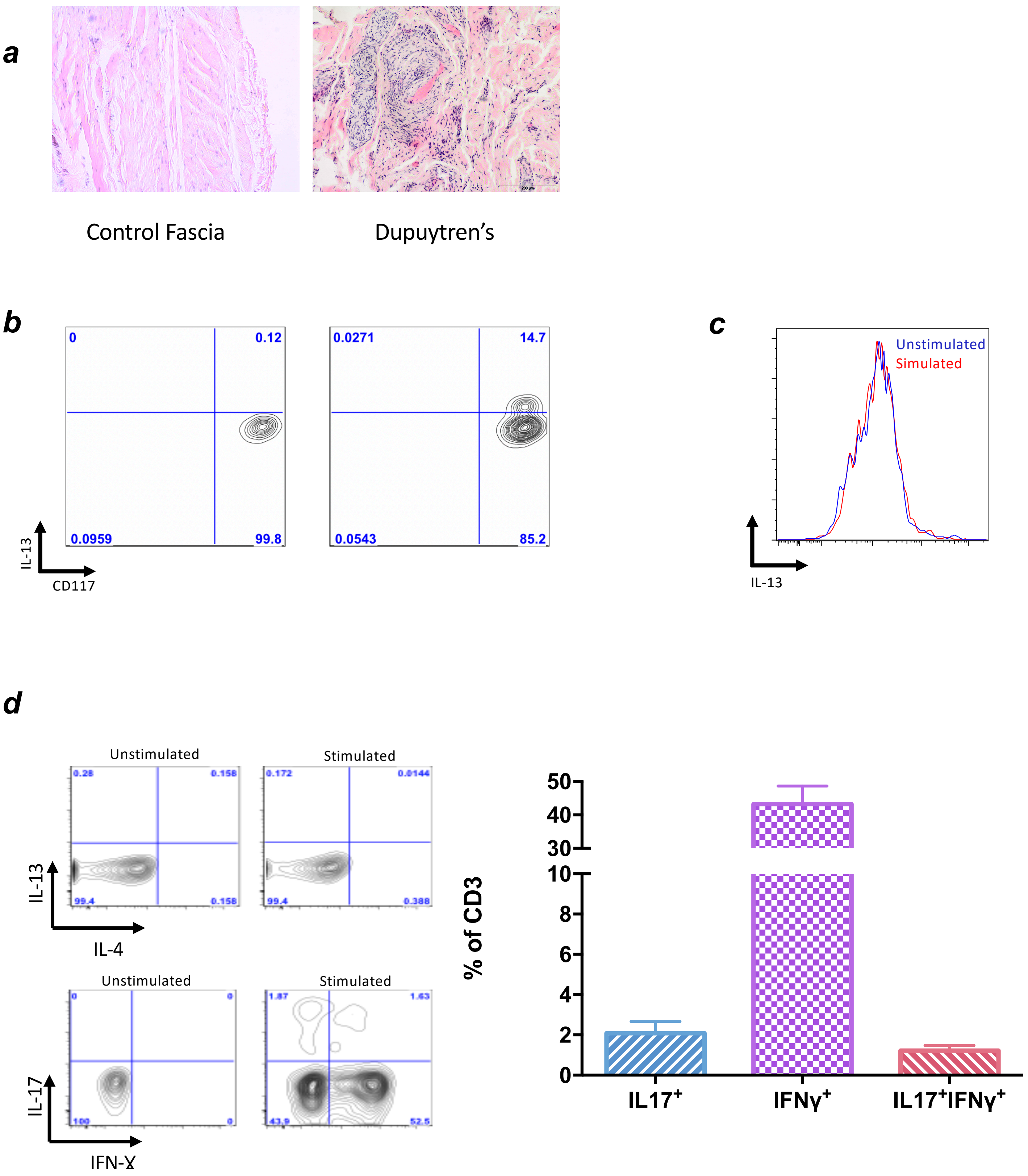

Supplementary Figure 1. Inflammatory cells in Dupuytren's Disease. (A) Histological staining of control fascia and Dupuytren's tissue stained with H&E, at 10 ×magnification. (B) Representative intracellular flow cytometry plot of unstimulated and stimulated CD117+ mast cells, from disaggregated Dupuytren's tissue, stained with anti-IL13. (C) Representative flow cytometry histogram of intracellular IL-13 in unstimulated and stimulated CD64+ macrophages, from disaggregated Dupuytren's tissue. (D) Characterisation of T-cells in disaggregated Dupuytren's tissue. Representative flow cytometric plots of unstimulated and stimulated CD3+ T-Cells following intracellular staining with antibodies against IL-4, IL-13, IL-17 and IFN aplha. Graph displays the proportion of T-cells positive for IFN-alpha.

a

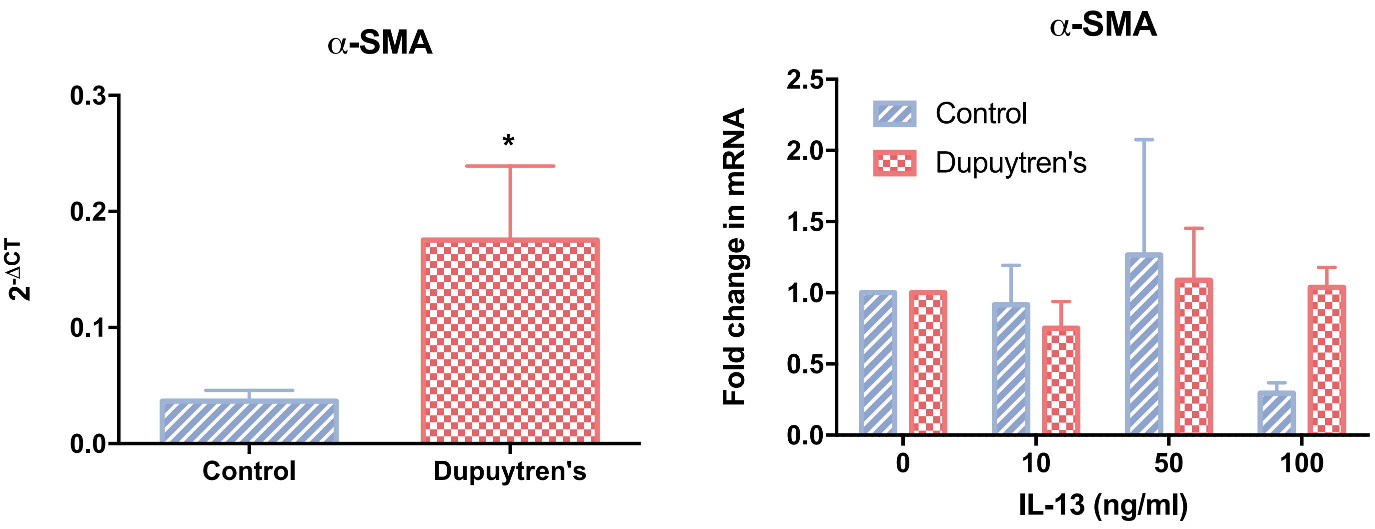

b

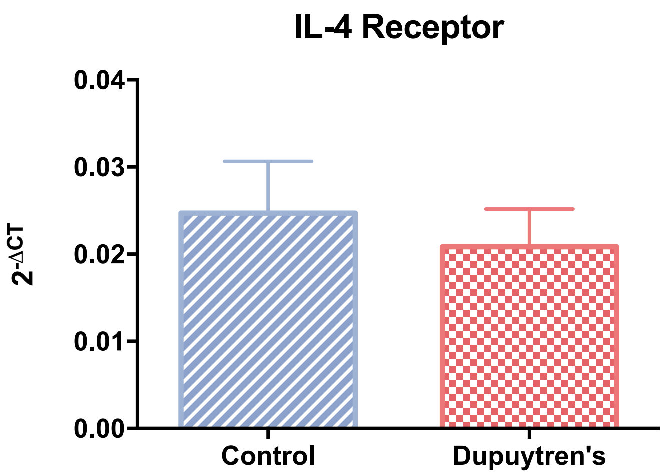

Supplementary Figure 2. (A) α-SMA expression in Dupuytren's disease. α-SMA expression in control fibroblasts and Dupuytren's myofibroblast, $2^{-\Delta CT}$ relative to GAPDH, mean ± SEM, n>5, *p<0.05. α-SMA expression in control fibroblasts and Dupuytren's myofibroblast following IL-13 treatment, n=6. mRNA gene expression expressed as fold change following normalisation to housekeeping gene (GAPDH) and then to relevant untreated cells. (B) IL-4 receptor expression in control fibroblasts and Dupuytren's myofibroblast, $2^{-\Delta CT}$ relative to GAPDH, mean ± SEM, n=6.

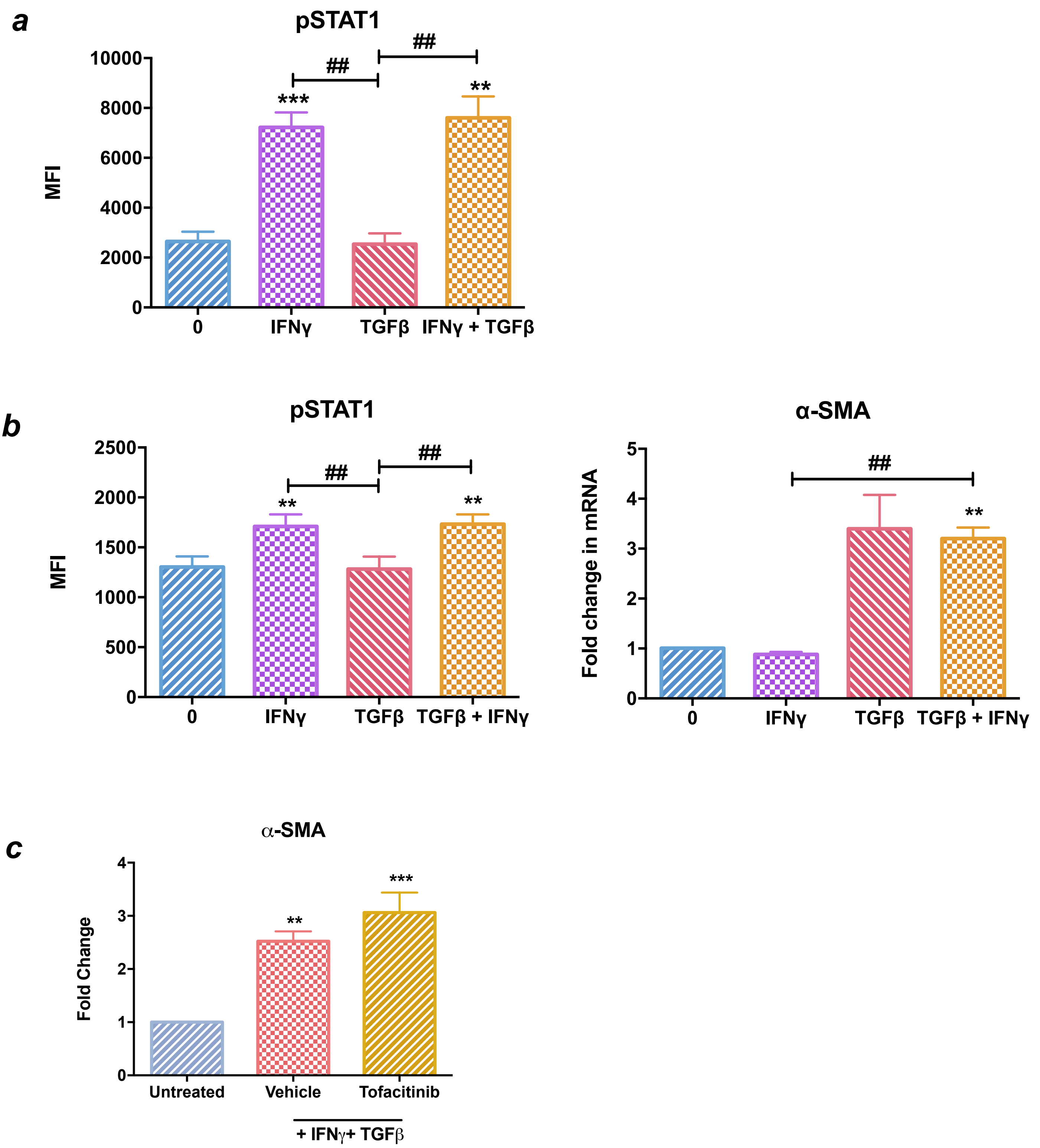

Supplementary Figure 3. IFNγ in Dupuytren's disease. (A) STAT1 phosphorylation and in human mast cells following IFN-γ and TGF-β exposure. (B) STAT1 phosphorylation and α-SMA gene expression in control fibroblasts following IFN-γ and TGF-β exposure. (C) α-SMA gene expression in control fibroblasts following IFN-γ and TGF-β exposure. Fibroblasts were pre-treated with tofacitinib or vehicle control (0.001% DMSO) for 30 mins prior to cytokine stimulation. All results are mean ± SEM, mRNA gene expression expressed as fold change following normalisation to housekeeping gene (GAPDH) and then to relevant untreated cells, n>4, * indicates significant difference from untreated cells, *p<0.05, **p<0.01, ***p<0.001, ****p<0.001. #p<0.05, ##p<0.01, ###p<0.001, ####p<0.0001.
